# Identifying protective *Streptococcus pyogenes* vaccine antigens recognized by both B and T cells in human adults and children

**DOI:** 10.1038/srep22030

**Published:** 2016-02-25

**Authors:** Rasmus Mortensen, Thomas Nørrelykke Nissen, Sine Fredslund, Ida Rosenkrands, Jan Pravsgaard Christensen, Peter Andersen, Jes Dietrich

**Affiliations:** 1 Statens Serum Institut, Department of Infectious Disease Immunology, Denmark; 2 Department of Immunology and Microbiology, University of Copenhagen, Denmark; 3 Department of Pediatrics, Copenhagen University Hospital, Hvidovre, Denmark

## Abstract

No commercial vaccine exists against Group A streptococci (GAS; *Streptococcus pyogenes*) and only little is known about anti-GAS protective immunity. In our effort to discover new protective vaccine candidates, we selected 21 antigens based on an *in silico* evaluation. These were all well-conserved among different GAS strains, upregulated in host-pathogen interaction studies, and predicted to be extracellular or associated with the surface of the bacteria. The antigens were tested for both antibody recognition and T cell responses in human adults and children. The antigenicity of a selected group of antigens was further validated using a high-density peptide array technology that also identified the linear epitopes. Based on immunological recognition, four targets were selected and tested for protective capabilities in an experimental GAS infection model in mice. Shown for the first time, three of these targets (spy0469, spy1228 and spy1801) conferred significant protection whereas one (spy1643) did not.

Group A streptococci (GAS; *Streptococcus pyogenes*) are major human pathogens causing a wide variety of diseases ranging from uncomplicated infections like pharyngitis and impetigo to life-threatening invasive diseases^1^. A conservative estimate propose that GAS infections and their sequelae account for more than 500,000 annual deaths^2^. There are no licensed vaccines against GAS.

The most studied antigen is the surface M protein where both the variable N-terminal and its conserved C-terminal region have been proposed as vaccine candidates^3^,^4^,^5^,^6^. However, the presence of more than 220 different *emm* types^7^ and the extensive amount of evidence that immune responses against the M protein are associated with development of post-strep sequelae^8^,^9^ have made the investigation of conserved non-M protein antigens attractive. Some protective non-M protein antigens, such as the streptococcal C5a peptidase (ScpA), the IL-8 serine protease (SpyCEP) and fibronectin-binding proteins have been identified, although they have yet to enter clinical trials (reviewed in^10^,^11^).

The goal for this study was to identify new protective non-M protein vaccine candidates. The first selection criterion in our strategy was that the antigens should be upregulated following interaction with the host. The next requirement was an immunological recognition in human adults and children. Based on recent data, we decided that our vaccine candidates should be recognized by both antibodies and T cells. It is already well-established that antibodies have protective capacity^12^,^13^,^14^,^15^,^16^,^17^ but cellular responses have also been suggested to possess antibody-independent protective capacity in a murine GAS infection model^18^,^19^,^20^. In addition, in a recent study we showed that the majority of both children and adults not only possess antibody responses, but also strong Th1 responses against GAS antigens^21^. Besides immunological recognition, we also added the criteria that antigens should be conserved among GAS stains and be extracellular or associated with the surface of the bacteria.

Although our strategy in part overlaps with approaches used in previous GAS antigen discovery studies^22^,^23^,^24^,^25^,^26^,^27^, we succeeded in identifying three undescribed GAS antigens that were all recognized by T- and B cells. We show that the three antigens were able to protect against infection with GAS in a murine infection model.

## Results

### Selecting GAS antigens

The aim of this study was to identify and characterize protective GAS antigens that constitute both T- and B cell targets. Our approach was to select antigens that displayed increased gene expression during interaction with the host, as these are likely to represent key factors in the establishment of an infection. Several transcriptome studies of GAS bacteria recovered after interacting with the host have been published. These include experimental pharyngitis in cynomolgus macaques^28^, growth in human blood^29^, soft tissue infection in mice^30^ and phagocytosis by human polymorphonuclear leukocytes^31^. Among the upregulated genes in these studies we selected a subset that were all *i)* conserved with over 90% identity in most of the 26 (gap-free) GAS genome sequences that were available at the time of the study (representing 16 *emm* types, see Supplementary table 1) and *ii)* predicted to have an extracellular location with the pSORT v3.0 online software or to be integral to the membrane with extracellular domains using the TMpred server^32^. In total, we selected 21 GAS antigens (table 1) and all of these were expressed as recombinant proteins. An overview of the individual design for each recombinant antigen can be found in Supplementary table 2.

### Antigens recognized by human IgG

**Figure 1:**
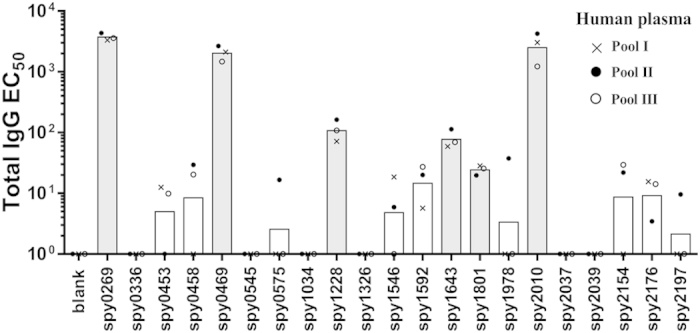
Initial screening of antigen specific IgG in human plasma pools. Total IgG specific for each of the 21 antigens was measured by ELISA in three plasma pools (10–12 donors in each pool). Bars represent geometric means of the EC_50_ calculated by a sigmoidal fitting of data points from a 10-fold plasma dilution series. Responses under 1 or too low for accurate fitting were adjusted to EC_50_ = 1. Grey bars indicate the antigens that were selected for IgG responder frequency analysis in individual donors.

All antigens were next tested for recognition by antibodies and T cells in humans, starting with recognition by IgG in human plasma. We first performed an initial screen for IgG reactivity in three pools of human plasma from 32 healthy adults (10–12 adults in each pool). This provided an indication of the “immune reactive” antigens, from which antigens would be selected for a more detailed analysis in individual donors. Spy0269, spy0469, spy1228, spy1643, spy1801 and spy2010 showed the highest IgG responses (Fig. 1, and Supplementary figure 1 that shows the plasma titration curves used to calculate the EC_50_ values). Spy2010 (C5a peptidase; ScpA) and spy0269 are well-known protective vaccine antigens^23^,^33^, but the other antigens were undescribed as protective antigens in the literature, and were therefore selected for further analysis in individual plasma samples. Spy0269 was included for comparison.

**Figure 2:**
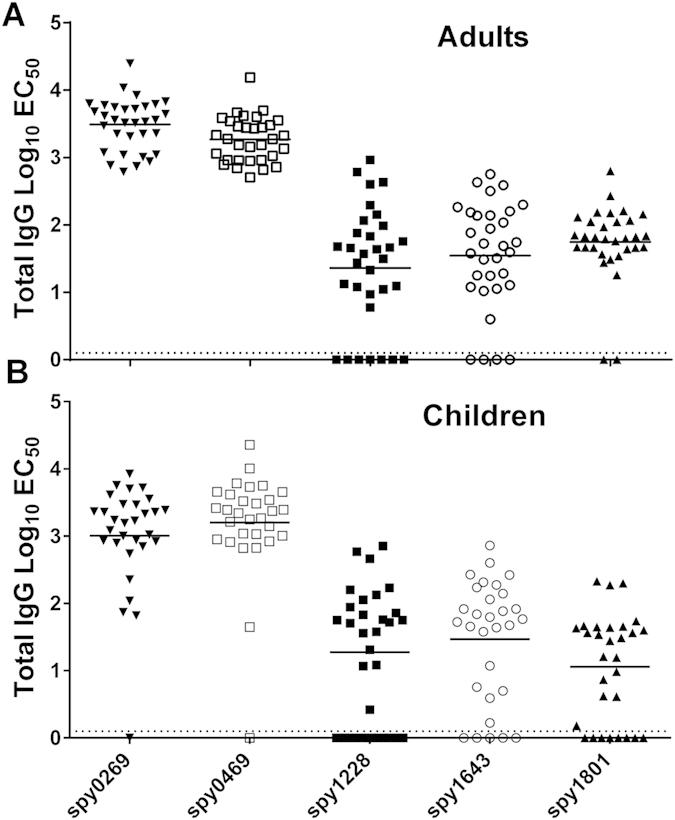
IgG responder analysis of selected antigens. Total IgG specific for selected antigens was measured by ELISA in plasma from (**A**) adults (n = 32) and (**B**) school aged children from 5–15 years (n = 30). Lines represent medians of the EC_50_ calculated by a sigmoidal fitting of data points from a 10-fold plasma dilution series. Responses too low for accurate fitting were adjusted to EC_50_ = 1. Responses for spy1801 have been reported for this donor panel in another publication^21^, but plasma samples have been re-run for this antigen along with the other antigens in this study for direct comparison.

For the analysis of individual plasma samples, we included a recombinant tuberculosis antigen (ESAT-6) as a negative control, which was purified in the same way as all the other antigens. Responses for each donor were “baseline-corrected” for every antigen by subtracting the EC_50_ value of the control antigen from the EC_50_ value of the GAS antigens. We defined all donors that showed responses above the baseline for a specific antigen as “responders” (for that particular antigen). The results showed that the antigens with the highest response in the pooled samples, spy0269 and spy0469, also had the highest responder frequencies in individual donors. Thus, all donors showed high IgG responses against these antigens (Fig. 2A and Table 2). Although not at 100%, the responder frequencies of spy1228, spy1643 and spy1801 were also high (88%, 94% and 78%, respectively), confirming the immunogenicity of these antigens. For comparison, we also measured the antibody responses in 30 children from 5–15 years of age, and no difference in the recognition pattern was observed although the response frequency was slightly lower for some of the antigens (Fig. 2B and Table 2).

In summary, we identified five antigens that are frequently targeted by human IgG responses in adults and children by performing a pre-screen of 21 conserved antigens with pooled plasma samples, followed by analysis of selected antigens in individual donors.

### Antibody target validation and comparison with other GAS antigens

**Figure 3:**
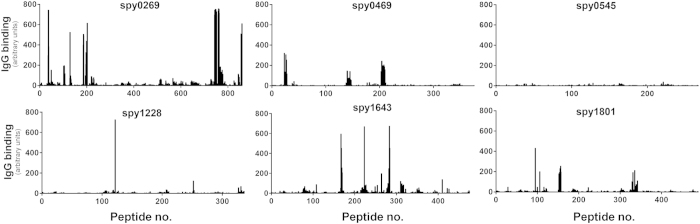
Antibody epitopes of selected antigens identified by peptide array technology. Binding of human IgG to 15-mer peptides overlapping with 14 amino acids on a peptide array spanning the entire length of each of the selected antigens. IgG binding was measured with a Cy3-conjugated goat anti-human antibody after incubation with plasma pooled from six adult donors. The black bars indicate fluorescence intensity for each of the peptides in arbitrary units that arise from summarizing over the R, G and B channels in the resulting image file. Peptides were randomly distributed on the array and data was mapped back in the right sequence order of the antigen after the experiment.

Measuring antibody responses against recombinant proteins expressed in *E. coli* involves the risk of measuring artifact responses against remaining contaminants from the purification process. Therefore, considering that most proteins contain both conformational and linear epitopes, the use of peptides to analyze antibody recognition (and avoid the problem with *E. coli* residuals) could validate that the antigens identified above were indeed targets for B cells in humans. As part of a separate ongoing antigen discovery project, we had access to peptide recognition data of all the targets selected above. These were measured by a recently developed high density peptide array technique where an array was synthesized spanning the entire length of all potential GAS proteins in the genome of the M1 SF370 strain with 15-mers overlapping with 14 amino acids (1aa spacing). This yielded a total of ~503,000 individual peptide fields from the 1,696 different protein sequences in the genome. The array was incubated with a pool of plasma from six human adults from the donor panel and after staining with a Cy3-conjugated goat anti-human detection antibody, linear peptides binding human IgG could be detected for each protein. Spy0269, spy0469, spy1228, spy1643 and spy1801 were all recognized in this assay (showing one or more peaks above 200–300 AU, which is 25–40% of highest possible readout), although spy1228 only showed one singular peak above 300 AU (Fig. 3). As expected, most regions in the antigens did not bind IgG. Setting a cutoff at 25% of the maximum readout, we defined the most immunogenic regions (“linear B cell epitopes”) for each protein (Supplementary table 3). In total we found 18 stretches of immunogenic amino acids in the five proteins with lengths from 15 amino acids up to as much as 32 amino acids (aa185–216 in spy0269). Except for spy0545 that is shown as a negative control, all the proteins had at least one linear B cell epitope.

A data comparison furthermore showed that spy0269, spy0469, spy1643 and spy1801 all belonged to the 20% predicted proteins in the GAS genome (349 in total) with at least one epitope with a signal strength >300AU (singular peaks omitted, data not shown). These 20% also included proteins with known protective capacity and for comparison a selection of these are shown in Supplementary figure 2 (showing several non-M proteins, as well as the M protein itself that displayed strong recognition in both the conserved and the variable region).

In summary, using a peptide array technology the five targets selected above were validated as being B cell targets in humans and spy0269, spy0469, spy1643 and spy1801 were furthermore found to belong to the 20% of the predicted GAS proteins that had at least one strong linear B cell epitope in this assay.

### Antigens recognized by cellular responses in humans

Having screened the selected proteins for antibody responses, we next focused on the recognition by T cells. T cells might contribute to immunity by amplifying secondary antibody responses^34^ and potentially also by eliciting antibody-independent protection^18^,^19^,^20^. We therefore tested all the antigens for antigen-specific cellular recognition in humans. We first performed a pre-screen of all antigens in a small set of donors, in order to identify the most immunogenic antigens for further analysis (in more donors). In this initial experiment we screened 18 adult donors for IFNγ release from PBMCs stimulated with the 21 different antigens. We chose IFNγ as we have recently shown this cytokine to be a sensitive indicator of cellular immunity in GAS exposed individuals^21^. Thus, most donors displayed cellular secretion of multiple cytokines, but IFNγ responses were by far the strongest, making this marker appropriate for cellular screening in this study.

**Figure 4:**
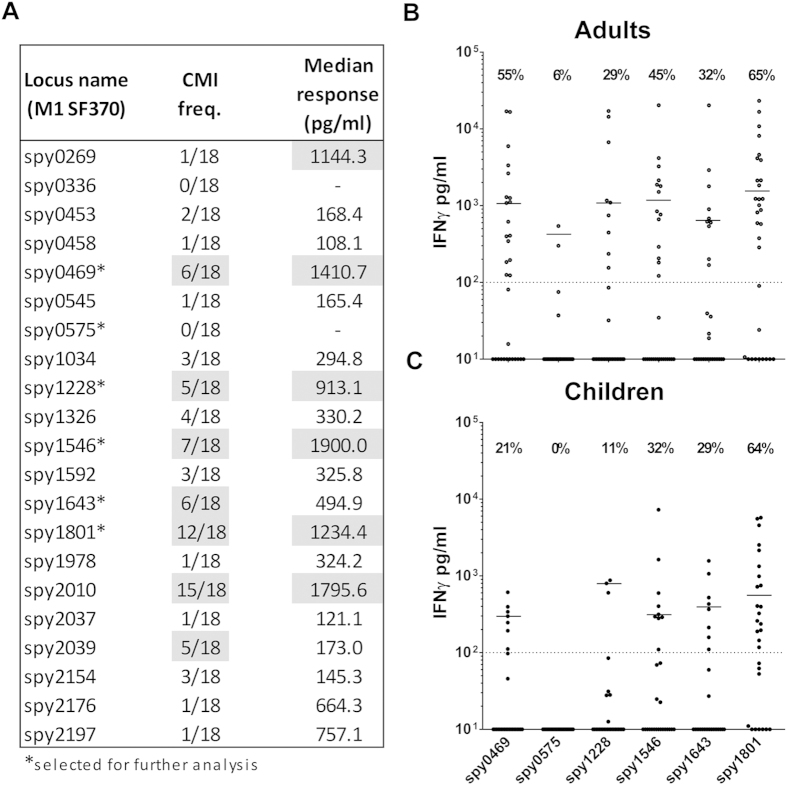
Screening of antigen specific IFNγ release in human PBMCs. IFNγ measured by ELISA in 7 day culture supernatants of stimulated PBMCs. (**A)** Pre-screen of the 21 different recombinant antigens in a smaller panel of adults (n = 18). Responder cut-off was set at 100 pg/ml. Gray marking indicate antigens with median responses over 500 pg/ml or over five responders. Five immunogenic antigens from this experiment plus a negative control were selected for further screening (marked by ‘*’). (**B**) Selected antigens were screened in an additional 13 adult donors (total n = 32) as well as (**C**) school aged children from 5–15 years (n = 28). Responses for spy1801 have been reported for this donor panel in another publication^21^. CMI; Cellular Mediated Immunity.

The results showed that the cellular immune response against GAS is distributed on multiple different antigens (Fig. 4A). Spy0469, spy1228, spy1546, and spy1643 showed both a high responder frequency as well as high median responses. However, cellular recognition was even more pronounced for spy1801 and spy2010. In contrast, Spy0575 and several other antigens showed low cellular recognition (Fig. 4A).

In the next analysis we included more adults as well as school-aged children from 5–15 years. We focused our analysis on five cellular targets identified in the pre-screen (spy0469, spy1228, spy1546, spy1643 and spy1801). Spy0575 was included as a negative control. A comparison of the IFNγ responses between adults and children showed that the responder frequency was similar for most of the antigens, although more adults responded to spy0469 and spy1228 compared to children (21% vs. 55% for spy0469 (p = 0.0198, Chi-square test) and 11% vs. 29% for spy1228 (p = 0.0809)), (Fig. 4B,C). Responder frequencies as well as the median responses for the adults were very similar to the pre-screening experiment, with spy1801 as the most immunogenic antigen. Responder frequencies ranged from 29% for spy1228 to 65% for spy1801 in adults and 11% to 64% in children. The total responder frequency on any antigen was 31/32 (97%) for the adults and 27/28 (96%) for the children.

Altogether, we evaluated 21 antigens for their cellular responses in humans measured by IFNγ and identified five highly immunogenic antigens with responder frequencies between 29–65% in adults and 11–64% in children.

### Protective efficacy of selected antigens

**Figure 5:**
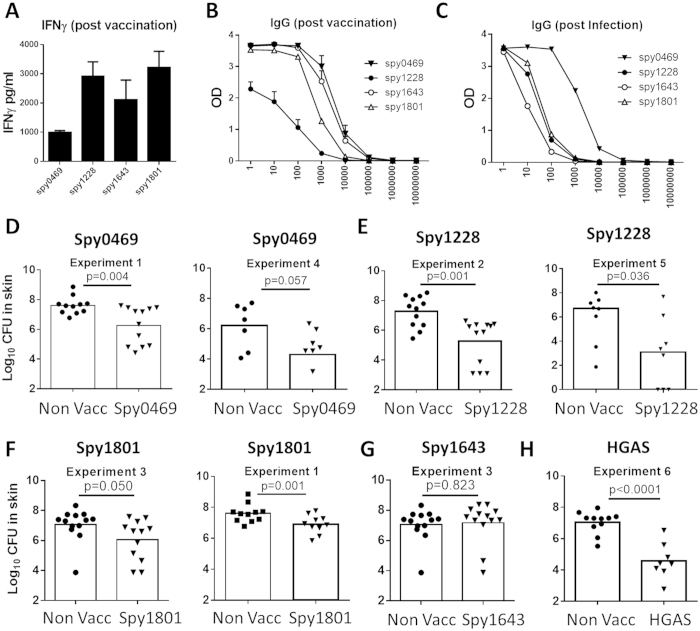
Immunogenicity and protective efficacy of selected antigens in the mouse model. (**A**) Female DBA/2 mice were vaccinated three times with indicated antigens. At week 2 post vaccination, PBMCs were stimulated with the vaccine antigen for 72 hours and secretion of IFNγ was analyzed by ELISA. (**B**) The serum IgG against the indicated antigens was measured by indirect ELISA. Antibody titers were measured by the reaction of a series of 10-fold dilution of sera with the antigen. The Antibody titration from the sera of 4 mice are shown. Bars indicate means ± SEM. (**C**) The serum IgG against the indicated antigens was measured by indirect ELISA using pooled sera taken from eight mice two weeks after a sublethal intraperitoneal GAS infection. (**D–G**) Mice were vaccinated three times s.c. with indicated antigens. Six weeks after the final vaccination, mice received an intradermal infection with 10^7^ GAS bacteria (MGAS5005). 4 days post infection the number of bacteria in a skin biopsy covering the entire affected area was determined. A t-test was used to calculate the p-value. All the antigens were tested in at least two independent experiments. For spy1228/spy1801/spy0469 results from two such experiments are shown.

Based on an overall assessment of the immune recognition data, we chose to evaluate the protective efficacy of spy0469, spy1228, spy1643 and spy1801 in a murine skin infection model. We chose this model, as we find it to be a particular robust model to screen antigens for their protective efficacy due to low variation and stable CFU levels, in particularly between day 4 and 7 post infection. As shown above, all the antigens were targets for both antibody- and cellular recognition in humans and so we first confirmed that the antigens were also immunogenic in mice. Mice were immunized s.c. with the antigens three times at two-week intervals. Two weeks after the final immunization, sera from individual mice were analyzed for IgG responses against the vaccine antigen. In addition, PBMCs were obtained from blood and stimulated *in vitro* with vaccine antigen for 72 hours, whereafter IFNγ was measured in the supernatants by ELISA. The results showed that all antigens were immunogenic and recognized by both cellular responses and antibodies (Fig. 5A,B). Furthermore, infected mice also developed IgG responses against all the selected antigens. Consistent with our observations in humans, the strongest responses were observed for spy0469 (Fig. 5C).

After confirming immunogenicity of the vaccine antigens in mice, immunized animals were infected by the intradermal (i.d.) route 6 weeks after the final immunization. Four days post infection skin biopsies from the infected area were analyzed for bacterial numbers. We chose day 4 because the infection in our mouse model peaks at day 4, whereafter it is gradually eliminated (data not shown). The results showed that spy0469, spy1228 and spy1801 all induced significant protection compared to non-vaccinated control mice (Fig. 5D–G). The protection (reduction in bacterial burden) varied slightly between the antigens with spy1228 inducing the highest level of protection, similar to the positive control which was heat inactivated GAS bacteria of a homologous strain (HGAS; Fig. 5H). In contrast, despite being recognized by both cellular and antibody responses in immunized and infected mice, spy1643 did not induce protection.

In summary, we were able to identify three potentially extracellular/surface exposed, conserved and immunogenic antigens. All of them represented B- and T cell targets in both humans and mice, and conferred significant protection in a murine GAS infection model.

### Analysis of cross-reactivity to human heart tissue

**Figure 6:**
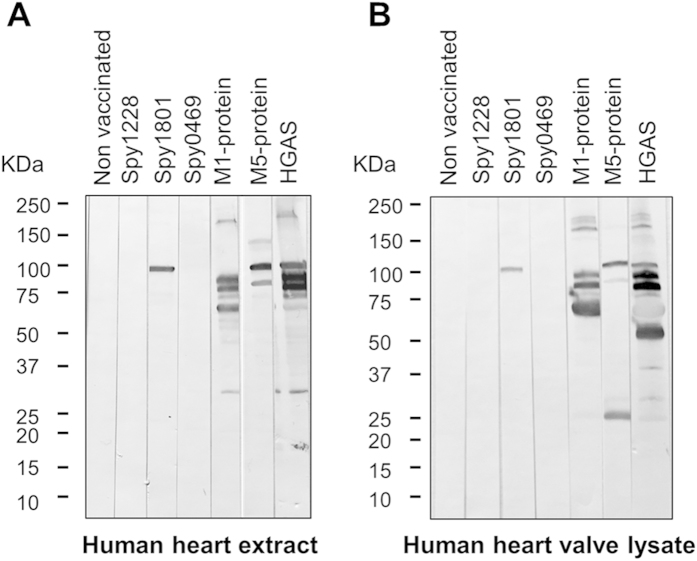
Cross-reactivity towards human heart tissue proteins. Anti-serum from mice vaccinated with the proteins indicated in the figure was used to test for cross-reactivity towards (**A**) human heart extract and (**B**) human heart aorta valve lysate by western blotting. The molecular weight markers in kilo Dalton (kDa) are indicated.

To address whether the identified GAS antigens might induce antibodies able to recognize human heart proteins, we tested murine anti-sera against each of the proteins for cross-reactivity to human heart extract proteins by western blots. As expected, anti-sera raised against the M1 and M5 protein as well as HGAS reacted with several proteins in both human heart extract as well as human heart valve lysate (Fig. 6A,B). In contrast, sera from spy1228 and spy0469 immunized mice did not react with any protein in the two different heart tissues samples, but surprisingly anti-serum against spy1801 reacted with a protein of approx. 100 kDa. A western blot with spy1801 anti-serum from another independent animal experiment confirmed this observation and we moreover validated the presence of recombinant spy1801 in the spy1801 batch used for immunization by mass spectrometry (data not shown). We also excluded that the cross-reactive band from spy1801 was due to *E. coli* contaminants in the Spy1801 batch used for immunization, since the spy1801 anti-serum did not react with any protein in *E. coli* lysates (data not shown). We thus conclude that, while spy0469 and spy1228 anti-serum does not react with proteins of human heart extract and heart valve lysate in a western blot, spy1801 anti-serum reacts with a protein of approx. 100 kDa.

## Discussion

This study describes the identification of three conserved antigens with protective capacity in an experimental GAS infection model. Various strategies have been employed during the last decade to identify protective non-M protein antigens from GAS^22^,^23^,^24^,^25^,^26^. Each of these studies identified one to nine protective antigens. Different technical approaches were adopted in these studies, which might explain why only a few antigens were identified in more than one study. Three such antigens were SpyCEP, ScpA and spy0269. Spy0269 was also shown in our study to be recognized by IgG in human plasma (Figs 1 and 2) and by our peptide array to contain several B cell epitopes (Fig. 3).

21 antigens passed our initial selection criteria. Six of these were recognized by cellular responses (spy0469, spy1228, spy1546, spy1643, spy1801 and spy2010) and six by antibodies (spy0269, spy0469, spy1228, spy1643, spy1801 and spy2010). Thus, there is a significant overlap between the targets of cellular and antibody responses. The same antigens were also recognized in infected mice suggesting that immunogenicity was independent of host species, and probably related to their expression and availability to the immune system. All individuals, except one child, exhibited adaptive immune responses to at least one GAS antigen, confirming that exposure to this pathogen is very common. In the i.d. infection model, immunization with spy0469, spy1228, and spy1801, but not spy1643, induced a significant reduction in the GAS colonization of the skin (Fig. 5D–G). This is, to our knowledge, the first time these antigens are described as protective antigens.

Spy1228 is a putative conserved lipoprotein^35^ with unknown function that has previously been shown to elicit an antibody response in mice and humans^22^. Extracellular location of this protein has also been verified experimentally^36^. Spy0469 showed strong antibody recognition in all adults and 97% children in our study as well as cellular responses in 55% of the adult donors compared to 21% of the children (p = 0.0198). The biological function of spy0469 is unknown but it contains a LysM domain that is used to locate proteins to the bacterial envelope via non-covalent linkage to peptidoglycan^37^. The extracellular location of this protein has also been verified experimentally^36^,^38^. Spy1801 does not have a confirmed biological function either, but it has been suggested to play a role in biofilm formation as its gene is highly upregulated during biofilm formation^39^. Consistent with that, computational comparisons indicate that spy1801 belong to a family of peptidoglycan hydrolases that includes a member in *Lactococcus lactis* that generates peptidoglycan disruptions that are important for biofilm formation^40^. Spy1801 (that is also annotated as “immunogenic secreted protein” (Isp)), is also known to induce antibody responses in humans^41^, so it was surprising that anti-sera raised against spy1801 cross-reacted with a 100 kDa protein in two different tissue extracts from the human heart. Although we cannot conclude that the epitope recognized by spy1801 anti-sera in the western blot is available for antibody recognition *in vivo* and thus play a potential role in GAS pathogenesis, it does raise a safety concern that should be further investigated.

The present work is the first to use a peptide array technology platform to identify linear B cell epitopes in the validation of GAS antibody targets. Using pooled serum from six donors, we confirmed the antigenicity of spy0269, spy0469, spy1228, spy1643 and spy1801 by identification of at least one linear antibody epitope. Subunit vaccines have an upper limit as to how many antigens that can be combined before recombinant expression of the final fusion protein become negatively affected by the molecular weight. However, numerous short linear epitopes like the ones identified in our study could potentially be included in a vaccine construct without obstructing recombinant expression due to size issues. This would allow the vaccine to target a large number of different GAS antigens. Given the high capacity of the array technology, all putative proteins in the GAS genome could be included in a single IgG epitope screen. By that, we were allowed to compare the signal strength of the epitopes from our selected targets with epitopes from every other possible protein in the M1 SF370 GAS genome. We found as much as 349 different proteins with at least one peak with signal strength >300 AU (data not shown). These 349 proteins constituted the top 20% of the genome and included spy0469, spy1643 and spy1801 as well as other antigens with published protective capacity (e.g. the M1 protein^3^,^4^, Streptolysin O^42^ and the streptococcal C5a peptidase^33^ (Fig. 3 and supplementary figure 2). This suggests that the peptide array technology can be used in future screenings for protective GAS epitopes, as it is a powerful tool to identify immunogenic antigens.

It should be noted however, that it is not fully known whether a selection strategy, based entirely on antigens/epitopes that are recognized during an infection, is the best strategy to discover protective antigens. Indeed, in several cases highly recognized epitopes (both T and B cell epitopes) may not be protective^43^,^44^,^45^,^46^,^47^ and it can be speculated that certain key antigens/epitopes have evolved to be weakly immunogenic (“cryptic”) in order to evade protective immune responses against them. In line with this, the highly protective hypervariable region of the M protein is weakly immunogenic in infected mice and humans, but immunization with the M protein in a strong adjuvant that can overcome the low immunogenicity results in protective immunity^13^. Interestingly, we found that antibody responses in humans against spy1228 were substantially lower than observed for spy0469 and spy1801. However, spy1228 showed the highest protection in immunized mice, indicating that high antibody titers in humans may not always be the best predictor of protective antigens, something that has also been showed for other infections^43^,^44^,^45^,^46^,^47^. In fact, inducing Ab response to cryptic epitopes has for viral pathogens been shown to result in increased protection^48^,^49^, and future Streptococcus antigen discovery strategies may consider this.

In summary, we have shown that the immune response against GAS includes both antibody and cellular responses against numerous different non-M protein antigens. Using immune recognition in humans as a selection criterion for evaluation in a murine skin infection model, we identified three protective non M-protein antigens.

## Methods

### Antigens

Full-length sequences of antigens upregulated in four microarray studies^28^,^29^,^30^,^31^ were obtained from *Streptococcus pyogenes* M1 SF370. Conservation of these antigens was evaluated by a BLAST search in a local database consisting of the 26 fully sequenced GAS genomes that were available at the time of the study (see Supplementary table 1). The genomes in the database were downloaded from the NCBI genome database (http://www.ncbi.nlm.nih.gov/genome/genomes/). Based on either being predicted to have an extracellular location with the pSORT v3.0 online software or to be integral to the membrane with extracellular domains using the TMpred server^32^, 21 conserved antigens were selected. Sequences of the 21 antigens were codon-optimized for expression in *Escherichia coli (E. coli)*. Transmembrane helices predicted by the TMpred server, were deleted for ease of expression and remaining fragments were linked by a sequence of -(GGGGS)_2_- (see Supplementary table 2 for detailed information on individual antigens). The sequences were made by chemical synthesis with an N-terminal His_6_-Tag followed by insertion into the commercial expression vector pJexpress 411 (DNA2.0). After transformation of *E. coli* BL21 AI (Invitrogen), protein expression was induced with 1 mM isopropyl β-D-1-thiogalactopyranoside (IPTG) and recombinant protein was purified as previously described^50^. Heat inactivated GAS bacteria (HGAS) were made by harvesting GAS colonies from blood agar plates in Tris-HCL buffer pH 7.5 before determining the bacterial concentration by plating. Bacteria were then diluted to 10^9^ CFU/ml and killed by heating the suspension for 120 min at 60 °C. As positive controls in western blotting experiments with human heart tissue samples, we also used recombinant streptococcal M proteins from M1 (SF370) and M5 (Manfredo), which were purified in the same way as described above.

The recombinant proteins selected for protection experiments were verified by Mass spectrometry after purification (data not shown).

### Human subjects

We enrolled two groups of volunteers; 32 healthy adults aged >20 years, and 30 school-aged children from 5–15 years as part of another recent study^21^. A simple questionnaire was used to screen the donors and all of them replied that they had not had a “sore throat” for at least 3 months before the blood samples were taken. The study was carried out in accordance with the regulations set forward by the Danish Ministry of Health and approved by the Committee on Health Research Ethics in the Capital Region (protocol no. H-2-2014-057) and the Danish Data Protection Agency (J. no. 2014-54-0733). Informed consent was obtained from all subjects.

### Lymphocyte cultures

PBMCs were purified from fresh heparinized blood using a density gradient. After washing, cells were incubated at 37 °C in round bottom 96-well microtiter plates (Nunc) in 200 μl serum-free AIM-V medium (Gibco; Invitrogen) containing 3 × 10^5^ human cells, or 2 × 10^5^ murine cells in 200 μl RPMI-1640 supplemented with 5 × 10^−5^ M 2-mercaptoethanol, 1 mM glutamine, 1% pyruvate, 1% penicillin-streptomycin, 1% HEPES and 10% fetal calf serum (FCS) (Gibco; Invitrogen). Recombinant antigens were used in concentrations of 5 μg/ml and as a positive control streptococcal Enterotoxin B was used in 1 μg/ml. Supernatants in duplicates or triplicates were harvested from cultures after 3–7 days for IFNγ ELISA.

### IFNγ ELISA

A sandwich ELISA was used to determine the concentration of IFNγ in culture supernatants as previously described for human samples^21^ and murine samples^51^.

### Detection of antigen-specific antibodies by ELISA

Maxisorp micro titer plates (Nunc, Maxisorp) were coated with the individual recombinant antigens in a concentration of 0.5 μg/ml and antibody levels in serum/plasma samples were determined by ELISA as previously described^21^. In brief, free binding sites were blocked with 3% skimmed milk (w/v) and plates were incubated with individual plasma samples in 10-fold serial dilutions starting with a 1:10 dilution. Antigen-specific IgG was detected with HRP-conjugated secondary antibodies which for human samples was polyclonal rabbit anti-human IgG (Dako) diluted 1:6000 or rabbit anti-mouse IgG (Zymed) diluted 1∶5000 for murine samples. Substrate was TMB-PLUS (Kem-En-TEC). Reciprocal serum dilutions corresponding to 50% maximal binding (EC_50_) were computed using the GraphPad Prism 6.04.

### SDS-PAGE and Western blot analysis

4–20% Mini-PROTEAN® TGX precast gels (Bio-Rad, Hercules, CA) were used, and 20 μg of human heart extract (sc-363763 from Santa Cruz Biotechnology, Dallas, TX) or human heart aorta valve whole tissue lysate (NB820-59218 from Novus Biologicals, Littleton, CO) were applied in each lane. Proteins were transferred to nitrocellulose by the Trans-Blot® Turbo™ transfer system (BioRad), the nitrocellulose was blocked in PBS with 5% skimmed milk, 0.1% Tween 20 and thereafter incubated with mouse serum samples diluted 1:200. Antigen-specific immunoglobulins were detected with alkaline phosphatase conjugated secondary antibodies.

### High-density peptide arrays of selected antigens

A peptide array spanning the entire length of all predicted proteins in the entire genome of the M1 SF370 GAS strain (1,696 predicted proteins) was synthesized with 15-mers overlapping with 14 amino acids (~503,000 individual peptide fields). The layout of the array was made with proprietary software using the FASTA sequence of GAS M1 SF370 as input. Peptides spanning human serum albumin, were also included on the array as a negative control protein that does not bind human IgG. All peptides were randomly distributed on the chip to reduce the possible effect of local area-specific noise. Arrays were made by Schafer-N® using maskless photolithographic synthesis adapted to solid phase peptide synthesis with the peptide C-terminal linked to the surface of the array. The microarray was incubated for 2 hours with a 1:200 dilution of pooled plasma from six randomly selected human adults. After washing the arrays were stained for 2 hours with 1 μg/ml of a Cy3-conjugated goat anti-human detection antibody. Images of stained arrays were recorded using an InnoScan900 micro array scanner (Innopsys) with an excitation wavelength of 532 nm and fluorescence intensity for each peptide field was calculated by summarizing over the R, G and B channels in the resulting image file.

### Bacterial strains and growth

GAS strain MGAS5005 (serotype M1) was grown at 37 °C with 5% CO_2_ in Todd-Hewitt broth (SSI Diagnostica) or on 5% blood agar (SSI Diagnostica) that was used as solid medium.

### Animal experiments

Female DBA/2 mice at 5–8 weeks of age were purchased at Harlan Laboratories (Horst, The Netherlands) and randomized to cages upon arrival. Handling of the animals was conducted in accordance with the regulations of the Danish Ministry of Justice and animal protection committees and in compliance with the EU Directive 2010/63/EU. The methods were carried out in accordance with the approved guidelines. All animal experiments were approved by an institutional animal committee. Mice were immunized subcutaneously (s.c.) at the base of the tail with 5 μg of recombinant antigen formulated in AddaVax^TM^ 1:1 (Invivogen). Three immunizations were performed with a two-week interval and four weeks after last immunization, mice received an intradermal (i.d.) injection of 0.5–1 × 10^7^ CFU (GAS strain MGAS M5005, M1 serotype). The mice were sacrificed 4 days post infection and skin biopsies were obtained by excising a specimen of 1–1.5 cm in diameter around the infected area. Vaccine-induced protection was evaluated by counting the CFUs obtained from plating suitable dilutions of skin homogenates.

### Statistics

CFU levels in challenged mice were compared by a student’s t-test in two independent animal experiments for each antigen. A chi-square test was used to compare responder frequencies in human adults and children. p < 0.05 was considered significant. All statistical analyses were carried out in GraphPad Prism version 6.04 (GraphPad Software Inc.).

## Additional Information

**How to cite this article**: Mortensen, R. *et al.* Identifying protective *Streptococcus pyogenes* vaccine antigens recognized by both B and T cells in human adults and children. *Sci. Rep.* **6**, 22030; doi: 10.1038/srep22030 (2016).

## Supplementary Material

Supplementary Information

## Figures and Tables

**Table 1:** Overview of selected *Streptococcus pyogenes* antigens.

Locus name (M1 SF370)	Lowest identity score^a^	Predicted cellular location^b^	Biological function^c^	References	Array^d^
spy0269	26/26 (>98%)	Cell wall	prgA; Putative surface exclusion protein	Galotta *et al.*, Infect Immun. 82(7), 2890–2901, 2014	i, ii
spy0336	25/26 (>99%)	Membrane	CsrS/CovS; two-component sensor histidine kinase	Levin *et al.*, Mol. Micro. 30 (1), 209-219, 1998	i, ii, iii
spy0453	25/26 (>99%)	Non-cytoplasmic	MtsA; metal ABC transporter substrate binding Lipoprot	Janulczyk *et al.*, Mol. Micro. 34(3), 596-606	ii
spy0458	25/26 (>98%)	Membrane	FtsK; DNA translocase		i, iv
spy0469	26/26 (>94%)	Non-cytoplasmic	Putative 42 kDa protein, surface antigen		ii, iv
spy0545	21/26 (>97%)	Membrane	Chromosome segregation ATPase		iv
spy0575	26/26 (>99%)	Membrane	Putative membrane spanning protein		i
spy1034	25/26 (>91%)	Membrane	Putative membrane associated protein		i, iv
spy1228	25/26 (>99%)	Non-cytoplasmic	Putative lipoprotein, nucleoside-binding protein		i, ii
spy1326	25/26 (>98%)	Membrane	Uncharacterized outer surface protein	Margarit *et al.*, *FASEB J.* 23, 3100–3112 (2009).	iv
spy1546	22/26 (>91%)	Membrane	Uncharacterized acetyltransferase		i, iv
spy1592	26/26 (>98%)	Non-cytoplasmic	Putative ABC transporter substrate binding Lipoprot.		ii
spy1643	26/26 (>97%)	Membrane	Uncharacterized protein		i
spy1801	25/26 (>92%)	Cell wall	isp; immunogenic secreted protein	McIver *et al.*, Infect Immun, 64(7), 2548-2555, 2014	ii, iii
spy1978	26/26 (>96%)	Membrane	Lrp; Leucine rich protein		i, ii
spy2010	24/26 (>95%)	Cell wall	ScpA; steptococcal c5a peptidase	O’Conner *et al.*, Infect Immun, 53(2), 432-434, 2086	i, ii
spy2037	24/26 (>99%)	Membrane	PrsA2; Foldase protein		i, iii
spy2039	26/26 (>97%)	Secreted	SpeB; Streptococcal cysteine proteinase	Elliott, S., J Exp Med. 81(6): 573 -592, 1945	i, iii
spy2154	26/26 (>98%)	Membrane	Uncharacterized protein		i, ii, iv
spy2176	26/26 (>92%)	Membrane	Uncharacterized protein		iii, iv
spy2197	25/26 (>97%)	Membrane	Putative membrane associated protein		ii, iv

^a^Checked in 26 different GAS strains with a local BLAST search.

^b^pSORT software v3.0 and Tmpred server.

^c^Inferred from UniProt.org.

^d^Array (i): Cynomolgus macaques [28], (ii): Blood [29], (iii): Soft tissue [30], (iv): PMN [31].

**Table 2:** IgG responder frequencies of selected antibody targets measured by ELISA in both adults and children from 5–15 years.

Locus name (M1 SF370)	Adults		Children	
	Responder frequency	Median Log_10_ EC_50_	Responder frequency	Median Log_10_ EC_50_
spy0269	32/32 (100%)	3,55	29/30 (97%)	3,23
spy0469	32/32 (100%)	3,28	29/30 (97%)	3,37
spy1228	25/32 (78%)	1,66	21/30 (70%)	1,76
spy1643	28/32 (88%)	1,73	25/30 (83%)	1,84
spy1801	30/32 (94%)	1,82	21/30 (70%)	1,56
