# Supplementary material for: Identifying protective Streptococcus pyogenes vaccine antigens recognized by both B and T cells in human adults and children
Source: Sci Rep. 2016 Feb 25;6:22030. doi: 10.1038/srep22030 (PMC4766568; doi:10.1038/srep22030)
Supplement: Supplementary Information [file srep22030-s1.pdf]

Supplementary Information

Identifying protective *Streptococcus pyogenes* vaccine antigens recognized by both B and T cells in human adults and children

Rasmus Mortensen[1,2*], Thomas Nørrelykke Nissen[3], Sine Fredslund[1], Ida Rosenkrands[1], Jan Pravsgaard Christensen[2], Peter Andersen[1], Jes Dietrich[1*]

[1]Statens Serum Institut, Department for Infectious Disease Immunology, Denmark
[2]Department of Immunology and Microbiology, University of Copenhagen, Denmark
[3]Department of Pediatrics, Copenhagen University Hospital, Hvidovre, Denmark

Supplementary Table 1. Genomic sequences used for analysis of sequence conservation for the GAS antigens in this study.

No. in database	Strain name	Type
1	SF370	*emm1*
2	MGAS5005	*emm1*
3	A20	*emm1*
4	M1GAS476	*emm1*
5	MGAS10270	*emm2*
6	MGAS315	*emm3*
7	SSI-1	*emm3*
8	MGAS10750	*emm4*
9	Manfredo	*emm5*
10	ATCC 19615	*emm5/49*
11	MGAS10394	*emm6*
12	MGAS9429	*emm12*
13	MGAS2096	*emm12*
14	HKU QMH11M0907901	*emm12*
15	HKU360	*emm12*
16	HSC5	*emm14*
17	MGAS8232	*emm18*
18	M23ND	*emm23*
19	MGAS6180	*emm28*
20	STAB901	*emm44*
21	1E1	*emm44*
22	NZ131	*emm49*
23	Alab49	*emm53*
24	MGAS15252	*emm59*
25	MGAS1882	*emm59*
26	7F7	*emm83*

Supplementary Table 2. Cloning strategy for the selected antigens of the study

**Locus name (M1 SF370)**	**MW** (kDa)	**Clonined fragment**
spy0269	94.7	aa33-849
spy0336	57.9	aa40-190
spy0453	34.4	aa21-310
spy0458	89.3	aa180-801, Δaa477-499
spy0469	41.1	aa24-389
spy0545	32.0	aa1-281, Δaa185-203
spy0575	31.3	aa91-271, Δ198-215
spy1034	29.6	aa1-263, Δaa103-121
spy1228	36.4	aa27-350
spy1326	41.2	aa1-360
spy1546	16.0	aa1-120
spy1592	53.6	aa26-380
spy1643	53.0	aa1-489, Δaa178-197
spy1801	54.5	aa25-503, Δaa251-270
spy1978	32.5	aa1-282, Δaa138-162, Δaa189-209
spy2010	122.3	aa90-1039, ΔAsp130, ΔHis193, ΔSer512
spy2037	34.4	aa27-309
spy2039	40.3	aa146-398, Cys192 -> Ser, His340 -> Gln
spy2154	32.5	aa128-292, Δaa155-173
spy2176	80.1	aa35-577
spy2197	37.9	aa1-341, Δaa181-200

Δ:deletions (replaced by -$(GGGGS)_2$-)

aa: amino acid number

->: amino acid substitution

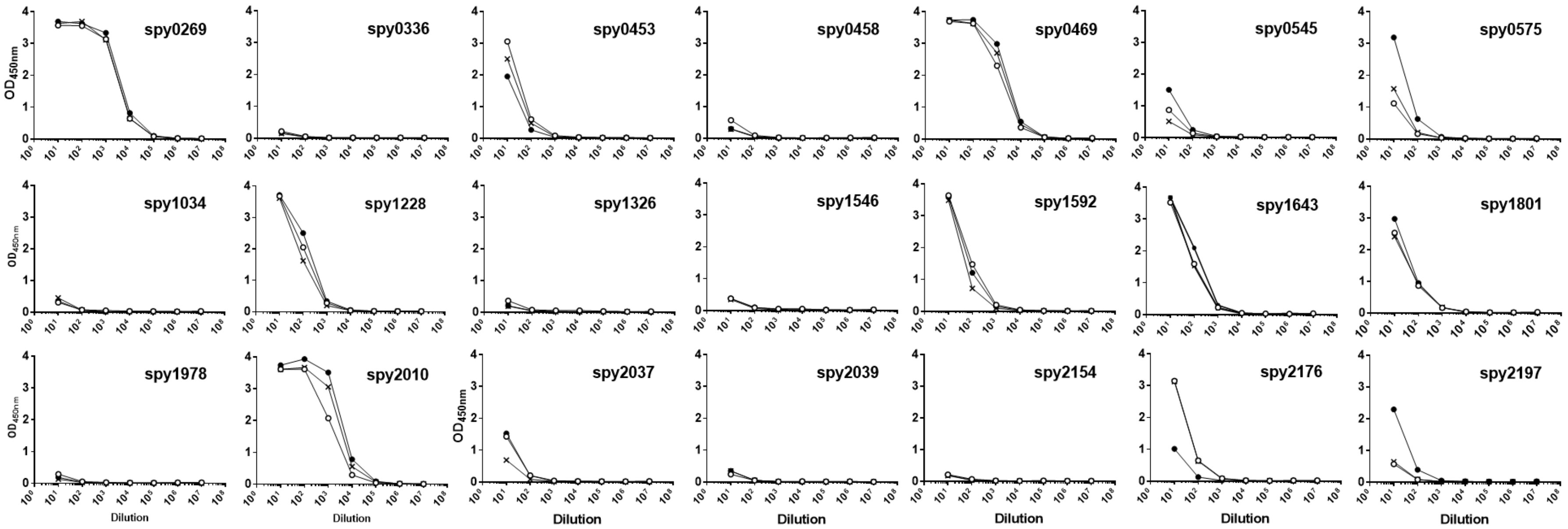

Supplementary Figure 1. Serum titration data for IgG reponses measured by ELISA

Antigen-specific IgG was determined by ELISA in three plasma pools from healthy adults (10-12 individuals in each pool). Symbols indicate individual data points for (×) pool I (●) pool II and (○) pool III

Supplementary Table 3. Immunogenic regions recognized by human IgG measured by a peptide array of 15-mers spanning the entire length of each antigen

Antigen	aa nr.	epitope sequence
**spy0269**	36-52	ADDRASGETKASNTHDD
	102-121	QDNEQKALTSAQEIYTNTLA
	129-149	AQGAEHQRELTATETELHNAQ
	185-216	QNIAKLNAMISNPDAITKAAQTANDNTKALSS
	743-765	TVPDLQVAPPLTGVKPLSYSKID
	758-782	PLSYSKIDTTPLVQEMVKETKQLLE
	856-873	VMLAAVGLTGFRFRKESK
**spy0469**	23-42	AQAQEWTPRSVTEIKSELVL
	140-161	ATPSDVPTTPFASAKPDSSVTA
	203-225	EPKTDISEAPTSANRPVPNESAS
**spy1228**	122-136	GVKFVIIDDIIEGKD
**spy1643**	167-184	SHHPAKQNTKKGWLIALF
	223-237	DSAKKATRLKAAAKA
	282-298	KAKVDSLKKAIAAITAV
**spy1801**	95-109	IGSSDNKAEAENQVD
	106-120	NQVDDKSTDHSKSTD
	151-171	RTPIQSLSPYVSDLDLSEIDI
	329-356	DTTGSGKRRAQIMEDLDKWIDDHGGTPA

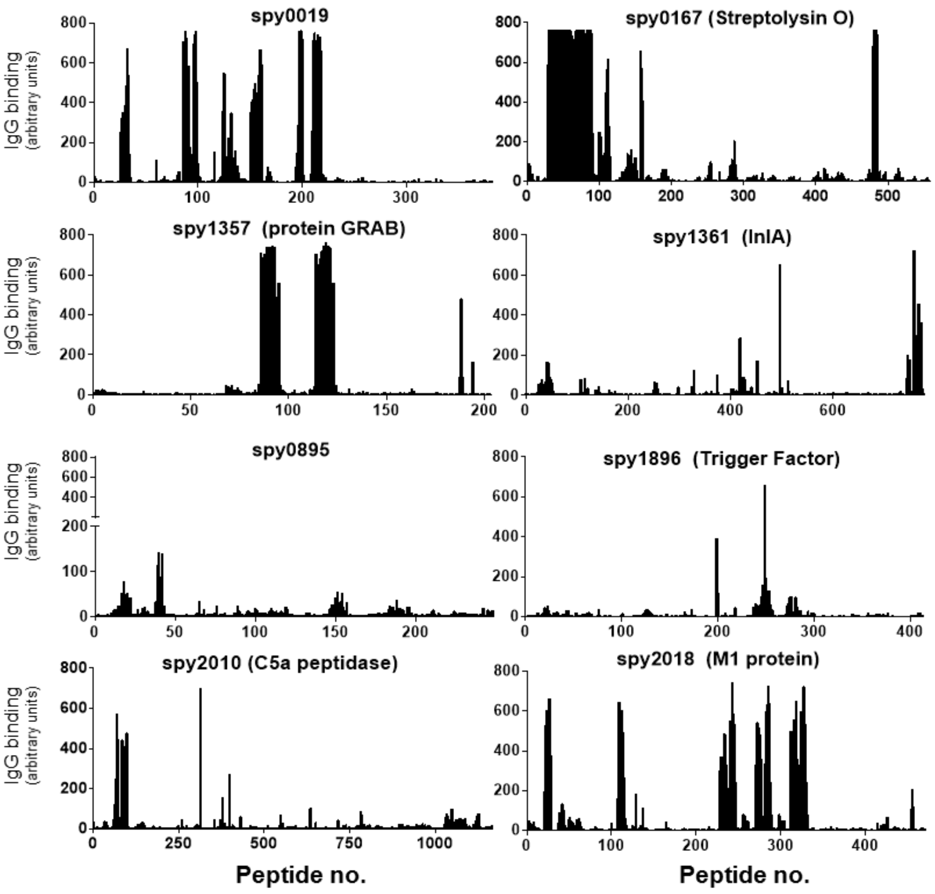

Locus name (M1 SF370)	Description	References
spy0019	Putative secreted protein	[24,25]
spy0167	Streptolysin O	[25,42]
spy0895	Predicted histidine protein kinase	[24]
spy1357	Protein G-related alpha 2M-binding protein; GRAB	[27]
spy1361	Putative internalin A (InlA)	[25]
spy1896	Trigger Factor	[26]
spy2010	Streptococcal C5a peptidase; ScpA	[33]
spy2018	M protein type 1	[3,4]

Supplementary Figure 2. Antibody epitopes of known antigens identified by peptide array technology.
Binding of human IgG on 15-mer peptides overlapping with 14 amino acids on a peptide array spanning every putative protein in the GAS M1 SF370 genome. Histograms are examples of known antigens. IgG binding was measured with a Cy3-conjugated goat anti-human antibody after incubation with plasma pooled from six adult donors. The black bars indicate fluorescence intensity for each of the peptides in arbitrary units that arise from summarizing over the R, G and B channels in the resulting image file. Peptides were randomly distributed on the array and data was mapped back in the chronological sequence of the antigen after the experiment. Spy0895 was included as an example of a known protective antigen, without an epitope >300AU.
